# Fragmentation of brittle material by shock wave lithotripsy. Momentum transfer and inertia: a novel view on fragmentation mechanisms

**DOI:** 10.1007/s00240-018-1102-6

**Published:** 2018-12-06

**Authors:** Othmar J. Wess, Juergen Mayer

**Affiliations:** Storz Medical AG, Lohstampfestrasse 8, 8274 Taegerwilen, Switzerland

**Keywords:** Lithotripsy, Shock waves, Fragmentation, Momentum transfer, Inertia

## Abstract

Shock wave lithotripsy is the only non-invasive stone therapy and in clinical use since 1980. In spite of decades with millions of patients treated, the mechanism of fragmentation is still under debate. Detailed knowledge of the fragmentation process is required for improvements regarding safety and efficiency. The purpose of this paper is to gain a deeper insight into the mechanism of fragmentation by drawing attention to basic physical laws of inertia and momentum transfer. Many fragmentation experiments are based on the overall efficiency of multiple shock waves in crushing kidney stones or artificial model stones utilizing small baskets or latex pouches. Due to the high dynamic nature of the fragmentation process, in vitro and in vivo, a detailed action of a single shock wave on a particular stone target is difficult to investigate. We utilized a bifilar stone suspension, which allowed us to observe horizontal movements of model stones, their return to the initial position and orientation for repeated exposure of separate identical shocks. The method does not describe the entire fragmentation process in detail but elucidates a mechanism, which may be effective throughout shock wave lithotripsy in general. Measurements on model stones in water revealed forces in the range of 370 N, acceleration values of 100,000–200,000 m/s^2^ (≈ 10,000 *g*) and gained momentum of 3.7 × 10^− 4^ kg m/s we consider sufficient to break most human urinary stones. Fracture patterns of repeated identical shock waves show typical features supporting spallation (Hopkinson effect) and the mechanism of momentum transfer. Schlieren and photo-elastic images provide a visual impression of spatial stress in a transparent acrylic glass cylinder, cavitation fields outside and at the surface of the cylinder, which are compatible with the inertia model. The proposed mechanism covers coarse as well as fine fragmentation. Collapsing cavitation bubbles may have an impact on the fragmentation process but although expected, a direct action of micro-jets on sample surfaces could not be detected.

## Introduction

Extracorporeal shock wave lithotripsy (SWL) dates back to 1980 when Chaussy [1] and co-workers treated first patients in Munich, Germany. To date, there are many lithotripter devices in the market featuring various shock wave generation systems with different shock wave field parameters. The fragmentation mechanisms are still under debate. Several mechanisms of stone comminution are reported such as tear and shear forces, spallation, quasi-static squeezing, cavitation and dynamic squeezing. Usually fatigue of internal stone structures is caused by application of numerous shock waves before stones break into small fragments. These mechanisms are comprehensively summarized and explained e.g. by Zhong [2] and in a consensus publication by Rassweiler et al. [3].

Tear and shear forces as well as spallation favour narrow focused shock wave fields to hit the target stones at the proximal stone surface. Quasi-static and dynamic squeezing (Sapozhnikov et al. [4]), require larger focal zones for lateral (circumferential) actions of shock waves perpendicular to the propagation direction. Cleveland and Sapozhnikov [5] developed a numerical model of elastic wave propagation in kidney stones. The theory of dynamic squeezing considers shear waves initiated at the corner of the stone reinforced by squeezing waves along the calculus.

The action is nutcracker-like and in combination with spalling a favoured mechanism of fragmentation [3]. The mechanism of cavitation is based on collapsing cavitation bubbles at the proximal and distal stone surface. According to Zhong [2] cavitation plays a major role in the second part of the fragmentation process. When coarse fragmentation has left larger fragments, cavitation is supposed to fracture them in to smaller particles. The mechanism of dynamic squeezing is considered the best theory to explain results of the numerical model [3, 4].

The conclusion of Rassweiler et al. is: *The new theories of stone disintegration favour the use of shock wave sources with larger focal zones* [3].

Experimental tests of the different mechanisms are usually performed under laboratory conditions on specific artificial stones featuring defined material parameters and spatial dimensions (cubes, spheres, cylinders etc.) The variability of natural stone types, sizes and compositions make a comparison of the efficiency of diverse shock wave parameters difficult. Teichman et al. [6] compared different lithotripter devices using a set of natural kidney stones in vitro and could evaluate the overall efficiency of the devices, but did not determine a favourable shock wave parameter responsible. In addition, the complex variability of treatment parameters makes clinical studies with different lithotripters, diverse stone populations and a varying treatment regime hardly comparable. Particularly treatment success and possible side effects depend on skills and treatment strategies, are dose dependent and have to be counterbalanced against each other.

Usually we find kidney stones trapped in renal calices, renal pelvis or ureter. They are imbedded in soft tissue or may be surrounded by liquids. They are softly fixed and can be slightly moved by external forces. Most fragmentation experiments mimic these conditions by using either small baskets or shock wave transparent latex compartments where stones may float and move a few millimetres and fragments re-collect at the bottom of the compartment to be ready for additional shocks. The overall fragmentation efficiency may be evaluated under these almost realistic conditions [6]. However, principle shock wave interaction with single shock waves cannot be clearly investigated since stones usually require several shocks to break. They change their location and orientation frequently unless they are fixed and unable to float.

This paper focuses on a physical mechanism of shock wave interaction with brittle material that, until now, gained little attention (Loske [7]) but may have significant impact on the fragmentation process. We experimentally investigated acoustic radiation force, or more specific, impulse and momentum transfer from shock waves to stone material by basic law of conservation of momentum.

## Materials and methods

### (A) Momentum transfer from shock waves to phantom stones

The mechanism of shock wave fragmentation was extensively explored with respect to pressure, wave propagation, squeezing, spallation, cavitation, focal size and others, but not to acoustic radiation force, or more specific, to impulse and momentum transfer. Whether or not an acoustic wave features a momentum at all, is positively answered e.g. by a paper of Schoch [8]. Müller [9], Loske [7] and Pye et al. [10] mention a momentum as a feature of shock waves but do not identify it as a significant mechanism of fragmentation.

The momentum of a shock wave is a vector quantity in the direction of propagation. We limit the calculation to the absolute value of a vector by considering only waves propagating normal to the surface and hitting the surface at the centre. Accordingly, the momentum density *P/A* of shock waves may be given by

$$P/A=\int\limits_{0}^{T} {p(A,t){\text{d}}t} \quad {\text{with}}\quad p={\text{pressure,}}\quad A={\text{cross}}{\kern 1pt} {\kern 1pt} \;{\text{section}}\;{\kern 1pt} {\text{area.}}$$

The momentum of a shock wave within a cross section area *A* may be given by

$$P=\int\limits_{A} {\int\limits_{0}^{T} {p(A,t){\text{d}}t{\text{d}}A} } .$$

**Fig. 1:**
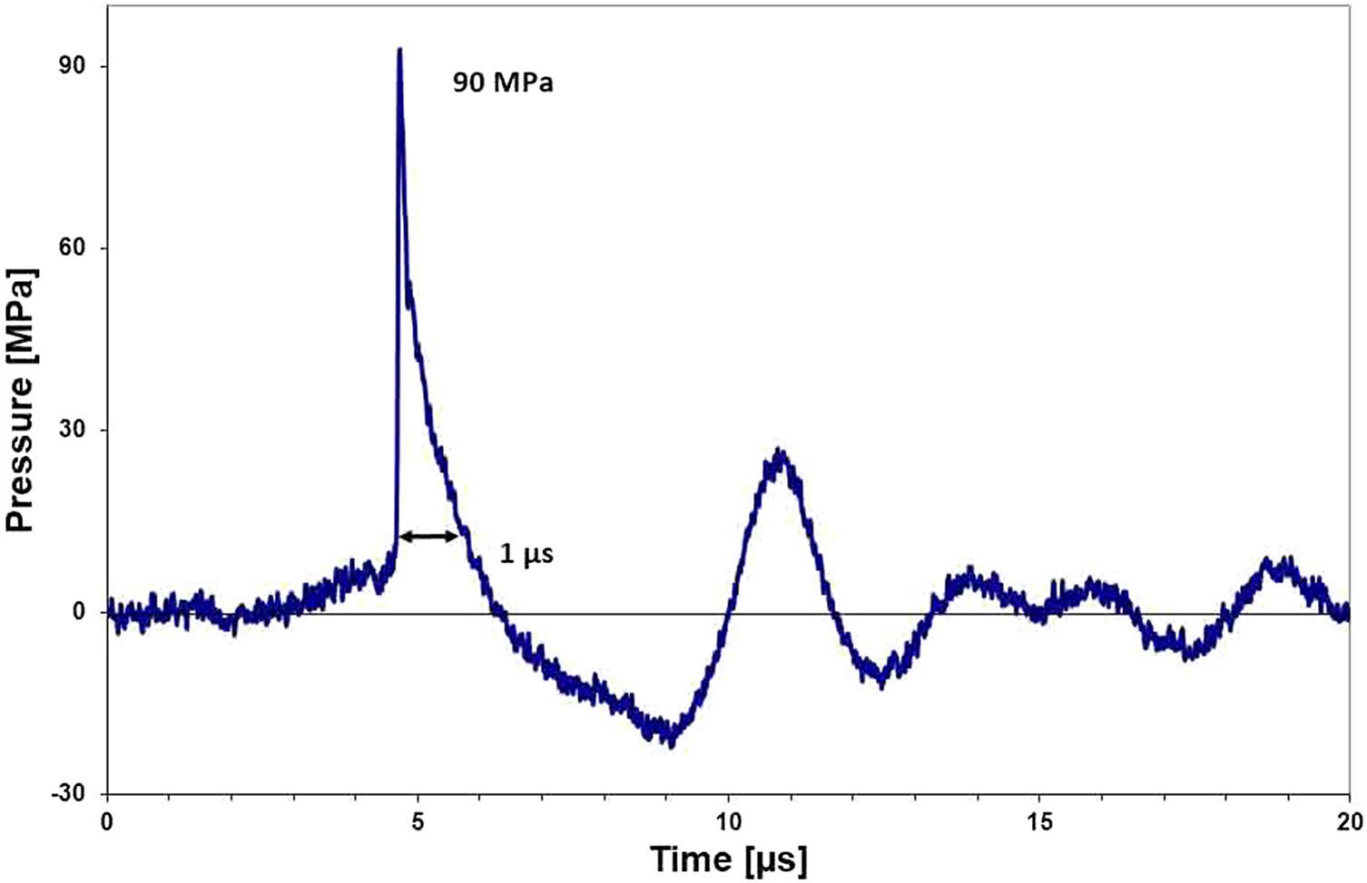
Shock wave profile with a positive pressure pulse of approximately 1 microsecond $\left(\mu \text{s}\right)$ duration (measured in accordance with IEC 61846, fibre-optic hydrophone). The − 6dB-lateral focus width is 3 mm

Figure 1 shows a typical pressure profile at the focus of a shock wave generator. Within the cross sectional area the peak pressure declines to the − 6dB-limit without changing the characteristic pattern of the pressure profile. We can separate the positive part of the pressure profile incorporating a positive momentum followed by the negative part with a negative momentum. It follows a smaller positive peak with a lesser positive momentum.

According to the different acoustical properties (*Z*_stone_ ≠ *Z*_water_, acoustic impedance *Z* = *ρ c* with *ρ* = density and *c* = acoustic propagation velocity) of stone material and water and depending on the angle of incidence, a part of the acoustic energy is reflected whereas another part is transmitted to the stone.

The goal of our experiments is to investigate this process in principle, identify the transmitted momentum *P*, the according force *F* and impulse (*F*Δ*t*) of a single shock wave acting on a stone.

We reduced the complexity of a shock wave fragmentation procedure by isolating single shock waves on a defined target, able to move and swing back in its initial position for an additional identical shock wave exposure. We suspended first, artificial stones (spheres; diameter 15 mm, made of Bego cement [11] with a weight of approximately 3.7 g and second, 1 cm^3^ cubes made of plaster of Paris with weight approximately 1 g) by two filaments (length approximately 20 cm, fixed in a distance of approximately 10 cm) in a water bath [12]. The bifilar suspension allows the stone to move horizontally if hit by the shock wave from lateral sites and return to its exact rest position due to gravity without losing its initial orientation. The stone was positioned centrally on the shock wave propagation axis (centre of gravity on the axis). The impinging shock wave front is almost parallel to the stone surface. This applies within the − 6dB-focal area (Ø 3 mm) and nearby for the cube and approximately also for the 15 mm sphere.

If the shock wave pulse hits the stone off-centre, rotational movements of the stone will consume part of the energy. Repeated shock waves hit the target stone always at the same position until coarse fragmentation into larger pieces changes geometrical conditions. This configuration does not cover the complex conditions of a real fragmentation process with stones being exposed in variable positions and surface areas; however, it allows measuring the momentum transferred to a linear motion of artificial model stones.

A typical time profile of the shock wave is shown in Fig. 1.

**Fig. 2:**
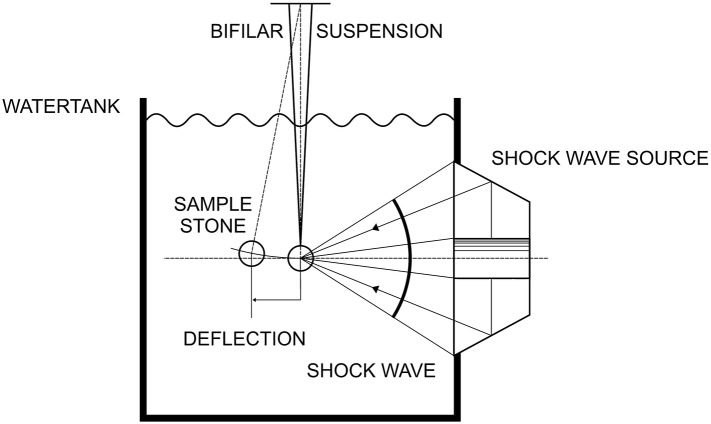
Experimental set up for shock wave based momentum transfer (schematic, water tank 45 × 45 × 60 cm)

Figure 2 shows the basic set up (schematic) with a bifilar suspension of a model stone in an open water basin and shock waves impinging from right lateral site.

Shock waves were generated by an electro-magnetic shock wave generator (Storz Medical Switzerland, focal depth 150 mm, aperture 178 mm, − 6dB-focus 3 mm, peak pressure *p*_+_ = 74 MPa, positive focus energy − 6dB-focus: *E*_+_ = 5 mJ, 5 mm-focus: *E*_+_ = 10.7 mJ, 5 MPa-focus: *E*_+_ = 80 mJ) mounted in a lateral wall of a water basin. The water was degassed and kept at room temperature (approximately 20 °C).

Recording the path-time-diagram of a stone movement, we can identify the momentum transferred from the impacting shock wave and the acceleration of the stone.

First series of experiments utilized 15 mm Bego spheres, weight 3.7 g in water. The shock wave impinged from right lateral side. Movements of the stone and fragments were recorded by a 25 Hz video mode of a commercially available video camera (Sony DV-Cam Model DSR-PD 150P, Japan).

A second series of experiment was done with 1 cm^3^ cubes of plaster of Paris (weight approximately 1 g) recorded by a 60 Hz video mode of a commercially available photo camera (D 800, Nikon, Japan).

### (B) Schlieren- and photo-elastic imaging

Shock waves are pressure waves and as such not visible to the naked eye. They are characterized by pressure variations and cause according density variation in media of propagation (water, tissue, solid materials). Schlieren-optical arrangements may visualize three-dimensional shock wave fields in water on a two-dimensional detector screen. Water is taken for its transparency for visible light and because the acoustic data of water are close to soft human tissue.

Schlieren-optics usually do not display pressure values directly but show gradients of pressure. Not only shock wave induced pressure gradients but also cavitation bubbles are visible simultaneously. This includes secondary (spherical) shock waves generated by collapsing cavitation bubbles.

**Fig. 3:**
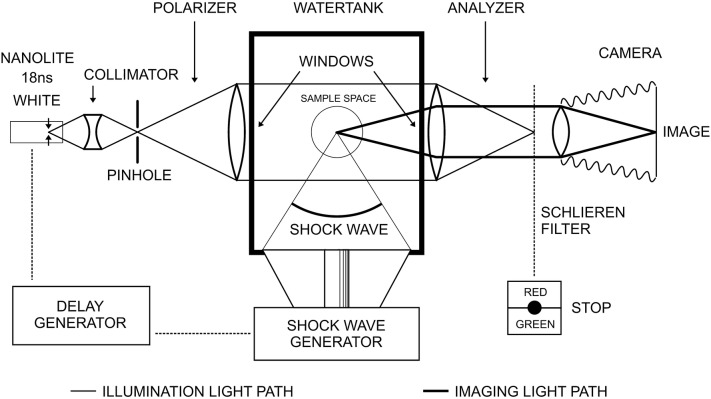
Schlieren-optical set up (schematic, water tank 45 × 45 × 60 cm)

The technique used is similar to the one published by Zhong [2] but instead of utilizing a monochromatic laser illumination this paper makes use of a special white light source (Fischer-Nanolite Nr. 151-KL-L, High-Speed Photo-Systeme, Wedel, Germany) with pulse duration of 18 nanoseconds (ns). Due to the white spectrum of the light source a special colour filter (Schlieren filter in Fig. 3) enables differentiation of positive pressure gradients (pressure rise) encoded e.g. in red versus negative gradients (pressure slope) encoded e.g. in green or vice versa. Without any colour filter, the arrangement can be used as “shadow optics” which displays strong pressure gradients as “shadow lines” on a bright background. A third version makes use of a circular light stop (Ø 5 mm) on the optical axis to stop all light originating from the point light source with the exemption of the light refracted by the pressure distribution of the shock wave. These images display pressure gradients as bright structures on a dark background. Combinations with colour filters are possible. The different techniques have specific advantages and are used in this paper whenever appropriate.

In order to freeze the fast travelling shock wave (velocity in water approximately 1500 m/s) the light is pulsed with a pulse duration of 18 ns. Choosing an appropriate delay between shock wave generation and light pulse we can vary the distance a shock wave travelled before it is frozen in the image.

Presupposed repeated shock waves are reproducible without modification—which is the case in an electro-magnetic generator—a sampled sequence of shock wave images can be taken and display the shock wave field at any point in time. In addition, the accompanying field of cavitation bubbles and the associated secondary shock waves can be recorded at the same point of time. The diameter of the circular shock waves divided by the propagation velocity directly identifies the time passed since the bubble collapsed. Figure 3 shows the basic Schlieren-optical set up.

Schlieren-optics and photo-elastic imaging can be applied simultaneously when e.g. translucent acrylic glass samples (acrylic glass cylinder or cubes) are placed between two inclined polarizers (polarizer and analyser). This technique allows for visualization of shock waves entering model stones made of transparent photo-elastic materials. The instant spatial distribution of strain and tension is displayed within a model configuration in colours.

## Results

### (A) Momentum transfer

The bifilar suspension allowed the spherical ball to move horizontally when hit by a shock wave impulse laterally. The viscosity of the surrounding water significantly attenuates the velocity of the stone. Only the initial velocity of the stone, immediately after impact of the shock wave, is relevant for the calculation of the acceleration. It can be extracted from the deflection-time diagram (see Fig. 5).

**Fig. 4:**
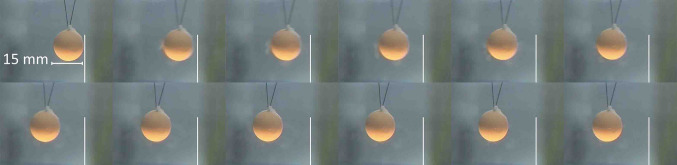
Lateral deflection of the stone pushed by a single shock wave from right. Series of 25 Hz video frames of a 15 mm Bego-stone with a weight of 3.7 × 10^− 3^ kg, time between frames 40 ms

Single frames of a 25 Hz video sequence (40 ms intervals) are displayed in Fig. 4.

The stone is accelerated within microseconds and retarded within 0.5 ms by the viscosity of the surrounding water. In spite of the slow video frame rate of 25 Hz the time history of the movement can be recorded.

**Fig. 5:**
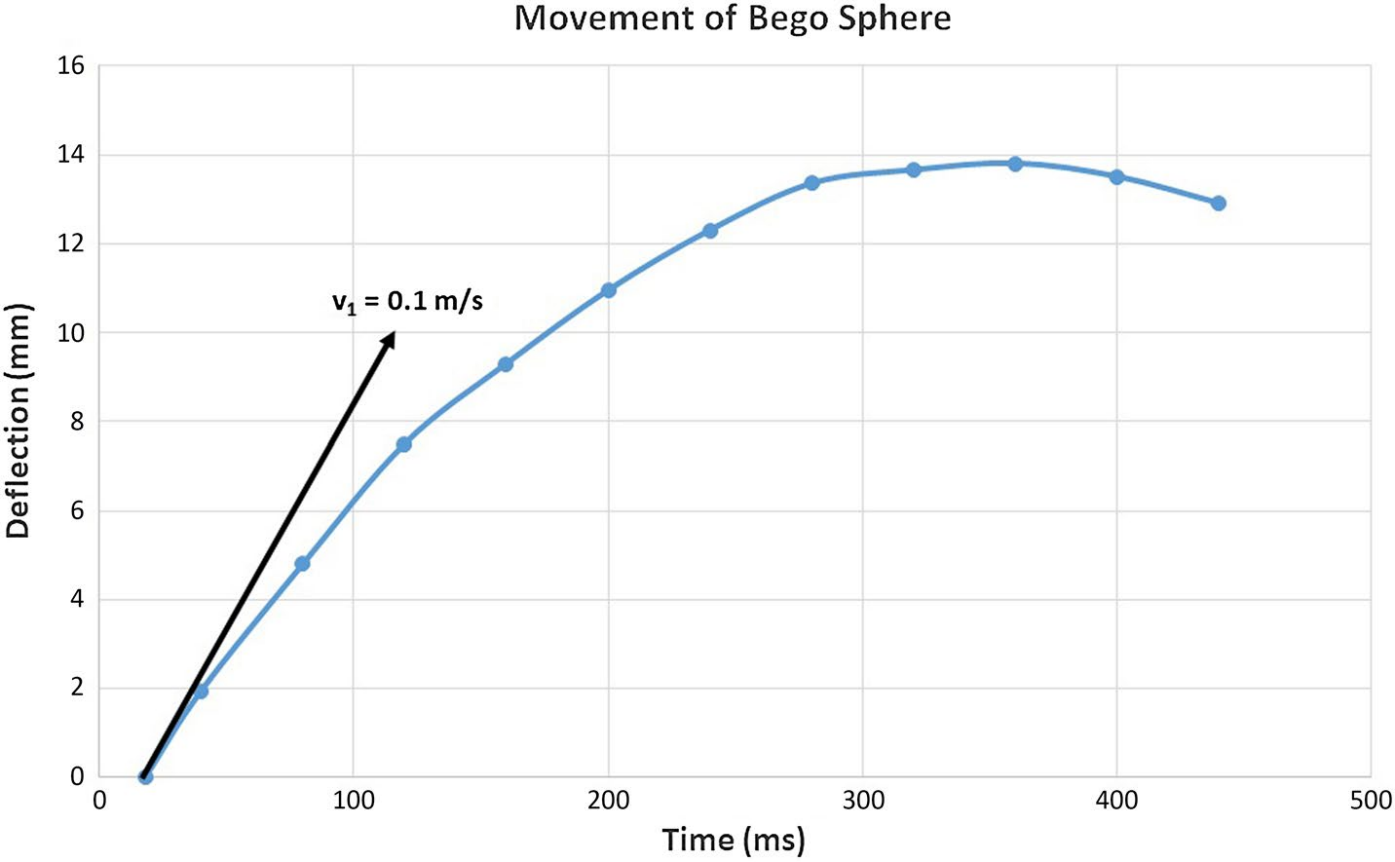
Deflection-time diagram

The according stone movement is shown in Fig. 5.

The slope of the curve defines the actual velocity of the stone.

Acceleration for the Bego-stone at the instant of shock wave impact is calculated as follows:

$${v_0}={\text{ }}0{\text{ m/s}}\;{\text{ (velocity before impact of the shock wave)}}~,$$

$${v_{\text{1}}}={\text{ }}0.{\text{1 m/s (velocity after impact)}}.$$

Momentum *P*_0_ before impact

$${P_0}={\text{ 3}}.{\text{7 }} \times {\text{1}}{0^{ - {\text{3}}}}{\text{kg }} \times {\text{ }}0{\text{ m/s }}={\text{ }}0.$$

Momentum *P*_1_ after impact

$${P_1}={\text{ 3}}.{\text{7 }} \times {\text{ 1}}{0^{ - {\text{3}}}}{\text{kg }} \times {\text{ }}0.{\text{1 m/s }}={\text{ 3}}.{\text{7 }} \times {\text{ 1}}{0^{ - {\text{4}}~}}{\text{kg m/s}}{\text{.}}$$

Acceleration time *t* = 1 µs (duration of the positive pressure pulse).

Instant acceleration:

$$a{\text{ }}={\text{ }}\Delta v/\Delta t~~~~~(\Delta v{\text{ }}={\text{ }}0.{\text{1 m/s}},~~~~\Delta t{\text{ }}={\text{ 1 }}\mu {\text{s}}),$$

$$a{\text{ }}={\text{ 1}}00,000{\text{ m/}}{{\text{s}}^{{\text{2}}~~~}} \approx {\text{ 1}}0,000g{\text{.}}$$

This compares to roughly 10^4^ times the acceleration of gravity *g*.

Accordingly, the effective force can be calculated:

$$F{\text{ }}={\text{ }}ma,$$

$$F{\text{ }}={\text{ 3}}.{\text{7 }} \times {\text{ 1}}{0^{ - {\text{3}}}}\,{\text{kg }} \times {\text{ 1}}{0^{\text{5}}}\,{\text{m/}}{{\text{s}}^{\text{2}}},~\quad ~F{\text{ }}={\text{ 37}}0{\text{ N}}{\text{.}}$$

The according impulse *I* (effective force *F* × duration of impact Δ*t*) is calculated by

$$I{\text{ }}={\text{ }}F{\text{ }} \times {\text{ }}\Delta t,~~~I{\text{ }}={\text{ 3}}.{\text{7 }} \times {\text{ 1}}{0^{ - {\text{4}}}}\,{\text{kg m/s}}{\text{.}}$$

The calculation only includes the positive (pushing) part of the pressure wave.

We assume that the following negative part of the pressure field has a retarding impact and does not compensate the pushing positive part as proven by the measurement of the bulk deflection of the stone. Nevertheless, there is a strong pulling force at the stone surface due to the negative portion of the impacting and reflected shock wave. The positive (pushing) part of the pressure field can reach pressure values in the range of 90 MPa and even higher, whereas the negative (pulling) part is limited by the dynamic tensile strength of water and its cavitation threshold.

The calculation is an estimation of the lower limit of acceleration forces since part of the fluid surrounding the samples is also accelerated. The additional accelerated mass (water predominantly in front and behind the stone) consumes part of the deployed shock wave energy but is not included in the calculation.

**Fig. 6:**
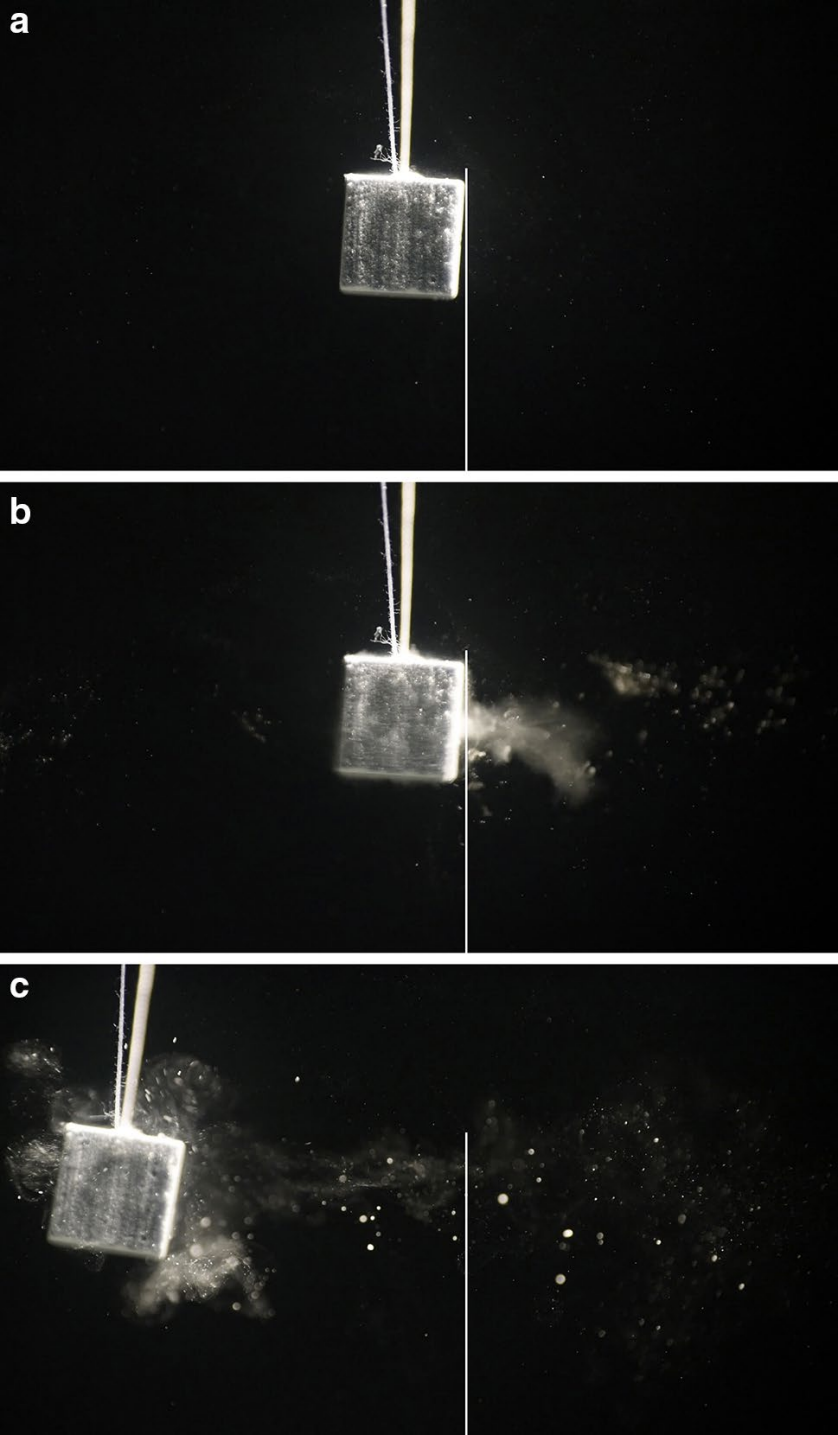
**a**–**c** Lateral displacement of a model stone by shock wave impact from right. Shock waves push the stone in the direction of shock wave propagation. Simultaneously small particles and dust are pulled from the surface and accelerated backwards to the shock wave source

Experiments with artificial 1 cm^3^ cube size stones (weight approximately 1 g) show a similar behaviour with respect to shock wave interaction (see Fig. 6a–c). Due to the lower weight, the initial velocity increases to approximately 0.2 m/s and acceleration is calculated to more than 200,000 m/s^2^ (≈ 20,000 *g*).

**Fig. 7:**
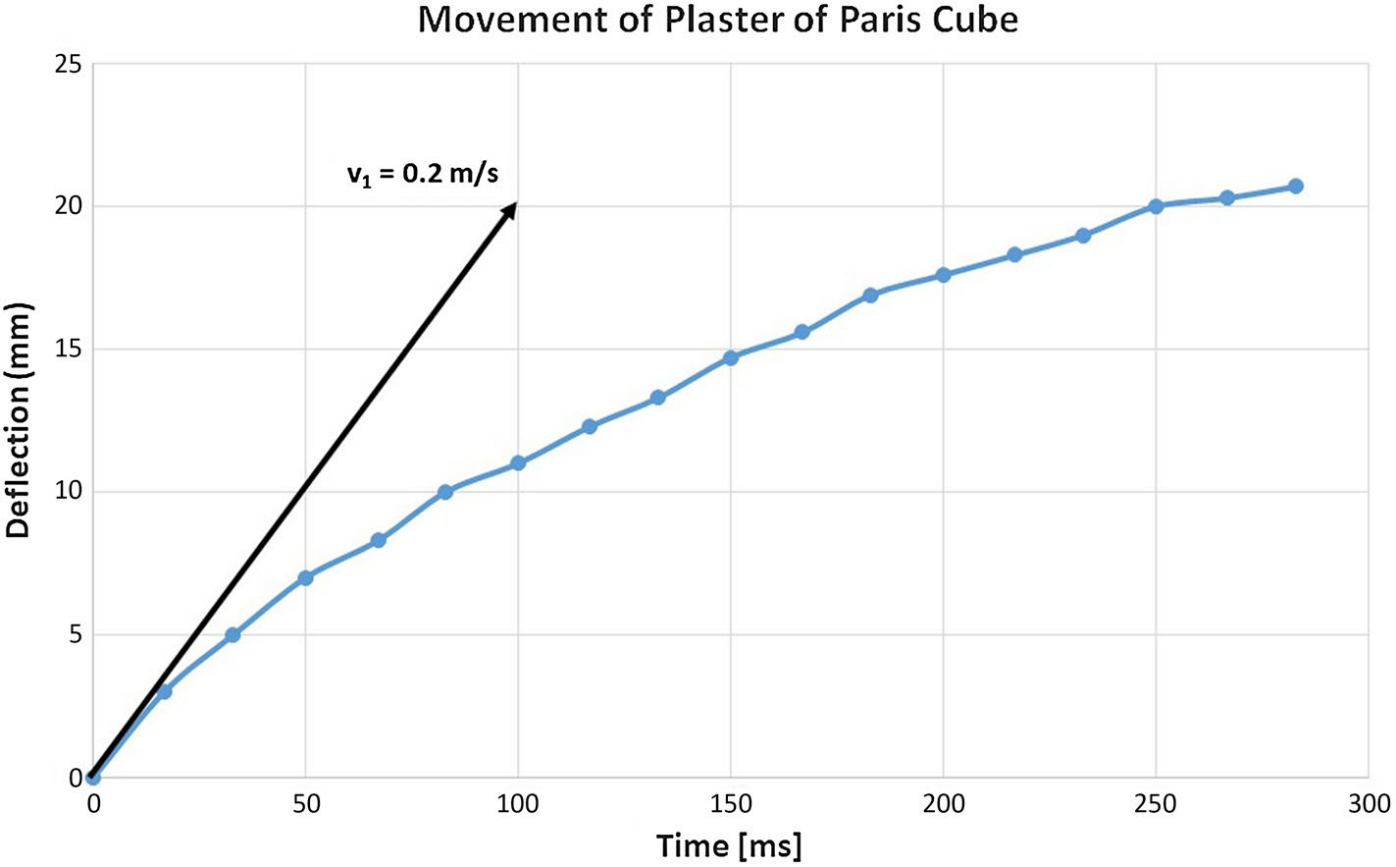
Deflection-time diagram

Figure 7 shows the deflection of the Plaster of Paris cube pushed by a single shock wave.

The documented deflection experiments show a typical behaviour for stones when hit by shock waves centrally (centre of gravity on the shock wave axis). Off-axis exposures consume part of the energy by rotational movements and hamper the evaluation of the momentum transferred by the impinging shock wave.

### (B) Phases of fragmentation

**Fig. 8:**
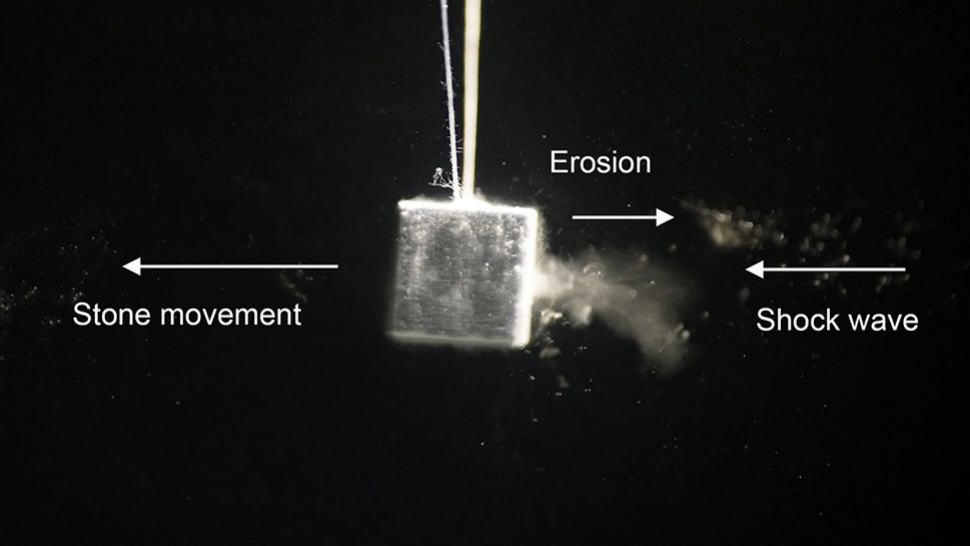
Erosion at front surface, first shock, shock waves from right (filaments are of equal diameter but are displayed differently due to the limited depth of sharpness of the photographic image)

**Fig. 9:**
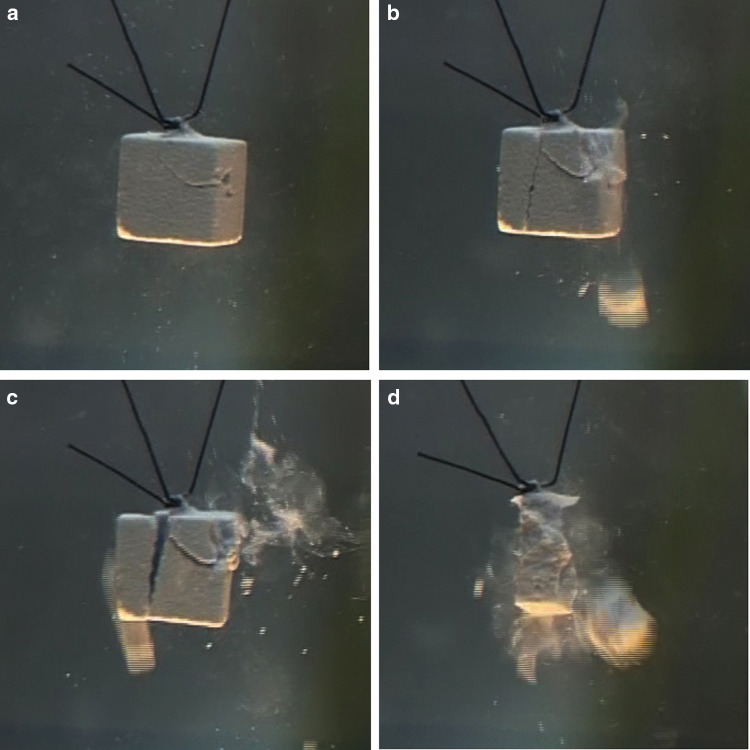
**a** Fracture lines at front corners, after ten shocks, shock waves from right. **b** Separation of front corners and fracture lines at rear surface (Hopkinson), after 15 shocks, shock waves from right. **c** Separation of rear surface layers at a distance of approximately 3 mm and further separation of front material, after 16 shocks, shock waves from right. **d** Multiple small fragments are generated after 23 shocks, shock waves from right

The fragmentation process shows typical patterns:

1. Immediately after impact of single shock waves (the focal peak centred on the right front surface) small particles are rapidly expelled from the surface and catapulted 1–2 cm to the reverse direction of shock wave impact. The intact stone has not yet moved as seen by the clear image of the filaments but is shattered as displayed by the slight blur (Fig. 8) compared to the clear image of the rest position of the stone in Fig. 6a.
2. Significantly slower than the front eruption of small particles and dust the intact stone starts to move in the direction of shock wave impact. Simultaneously, resisting inertia forces point contrary to the accelerating shock wave momentum and generate internal strain resulting in fracture lines (Fig. 9a). The pressure load of the impacting shock wave on the front surface is inhomogeneous and characterized by the lateral focus field. The focal peak at the centre starts to knock off a small crater out of the front surface.
3. Next shock waves shatter the stone and corners start to separate from the front surface.

Fissures at rear surface appear (Fig. 9b).

4. Spallation at the rear surface takes place in larger flat layers (approximately 3 mm) due to Hopkinson’s effect.

Single corners break out of the front surface (Fig. 9c).

5. Smaller, lightweight particles are pushed as a whole (Fig. 9d).
6. Repeated shock wave exposure results in many fragments smaller than the pulse-length of the impacting shock wave.

Depending on variations in stone materials, energy settings and accuracy of targeting, the number of shock waves required to create first fracture lines vary. The pattern of fragmentation, however, follows the course of action displayed in Fig. 9a–d and described above.

The experiment ends when the mass of the original stone diminishes by separation of larger fragments. This procedure differs from fragmentation experiments performed in small compartments keeping large and small fragments in place. In this case, the momentum of the shock waves affects the composition of small and large particles simultaneously. The mechanism of momentum transfer and impulse is effective also if impacted stones are slightly fixed and do not show a bulk motion. The pushing and pulling forces may act like a rocking motion or joggle.

The data, in particular the huge acceleration value of ≈ 100,000 m/s^2^ and the strong forces of ≈ 370 N highlight the short and powerful interaction of shock waves with brittle material. According to Newton’s third law the acceleration force exerts a force on a second body, the stone, of equal magnitude but with opposite direction (actio = reactio). These data exceed strain and stress limits of most natural kidney stones [12, page 79, Table VIII].

### (C) Schlieren- and photo-elastic imaging

**Fig. 10:**
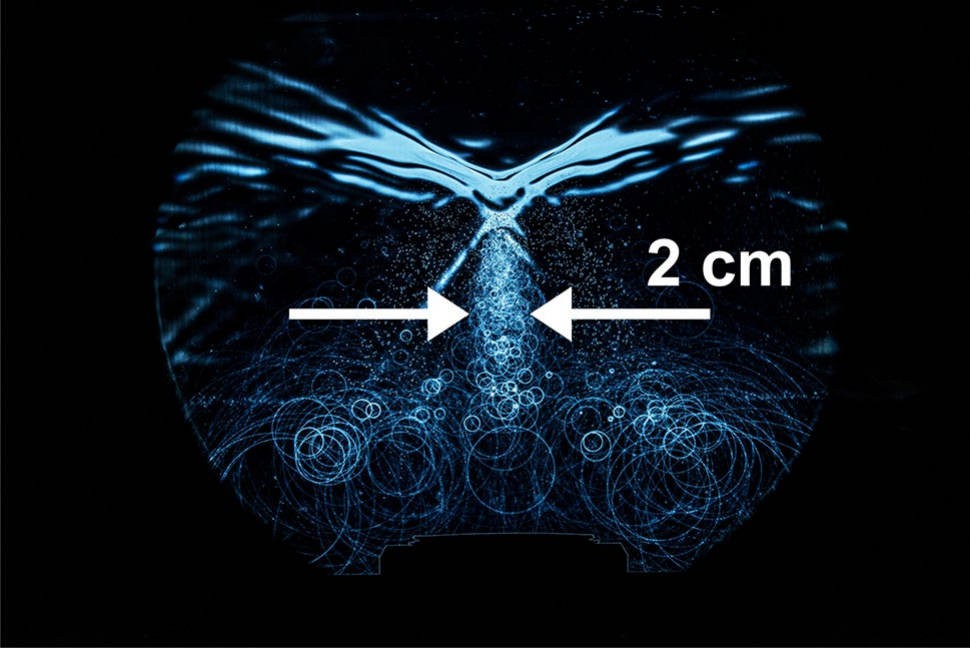
Shock waves travelling from bottom to top in a saturated medium with multiple cavitation nuclei

Short-term Schlieren- and photo-elastic imaging displays three-dimensional shock wave field characteristics in a two-dimensional plane. Depending on the cavitation features of the propagation, medium extended fields of cavitation bubbles can be visualized in different states of their lifetime. In Fig. 10, small bubbles are displayed immediately after generation by the passing shock wave, mature bubbles some microseconds later and collapsing bubbles radiating spherical secondary shock waves up to some 100 µs after the shock wave passed are shown in the bottom part of Fig. 10.

The central bubble trail in Fig. 10 is in the range of 2 cm, significantly wider than the 3 mm lateral − 6dB-focus extension and the pin-point-like erosion at the front surface (see Fig. 9a–c).

**Fig. 11:**
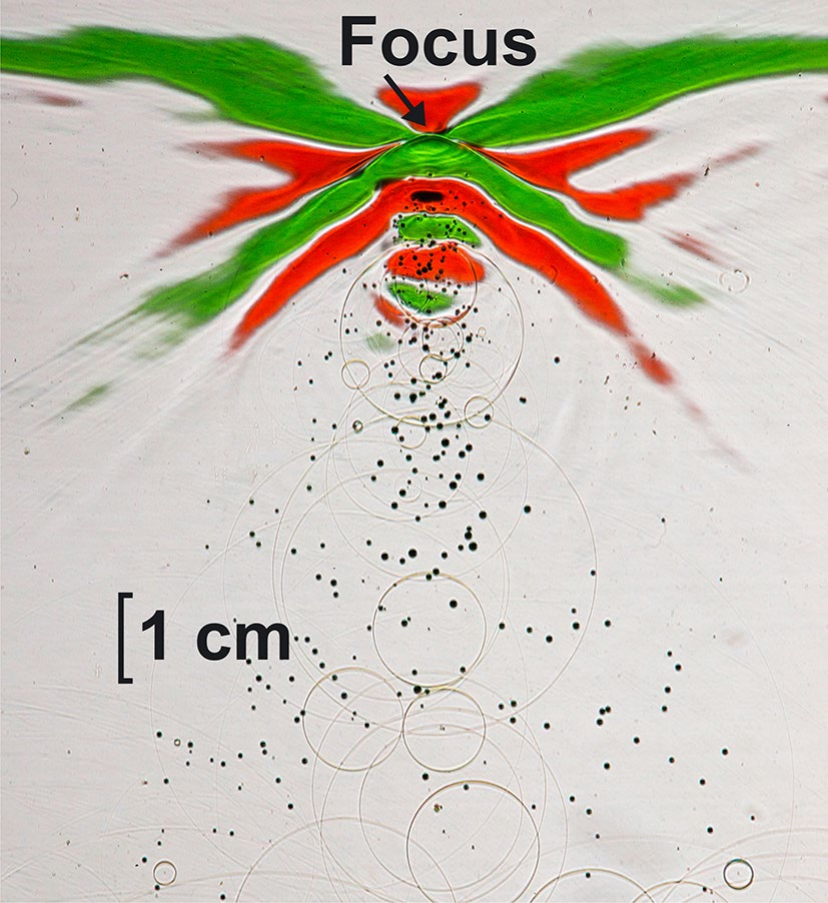
Shock waves close to the focus. Positive pressure gradient (rise) in red, negative pressure gradient (decline) in green, for further description see text. Shock waves travel from bottom to top

A closer look at the shock wave field near the focal zone is shown in Fig. 11.

Figure 11 shows a focussed shock wave close to the focal area. The shock wave travelled from bottom to top. The small red area in the centre displays the rapid rise of the pressure to approximately 90 MPa and determines the centre of the focus. The adjacent green area shows the descending slope of the pressure followed by a second rise (red) of the pressure distribution to second smaller positive peak. At the end of the green (descending) phase, small cavitation bubbles start to grow. The shock wave field is not limited to the small − 6dB-focus area (3 mm) but covers several centimetres in all three spatial dimensions. Since this is a snapshot, we can see the area the shock wave has passed some 10 µs before. Cavitation bubbles are generated at fixed locations when shock waves pass this position. They do not migrate but grow to diameters in the range of a millimetre. Some microseconds later, they collapse and radiate spherical shock waves. Time history of primary shock wave travel and following processes of growing and collapsing cavitation bubbles as well as of secondary shock wave propagation are displayed by spatial distances in the image.

No cavitation bubbles occur in front of the shock wave. The negative pressure starts to generate cavitation bubbles present as bubble trail behind the shock wave.

As shown in Figs. 10 and 11 the central bubble trail is significantly wider than the lateral − 6dB-focus extension. If cavitation would be the major mechanism of fragmentation, the whole front surface (Fig. 9a) would erode; Fig. 9a, however, displays a pin-point-like focused action well in accordance with the small focal dimensions of the shock wave.

**Fig. 12:**
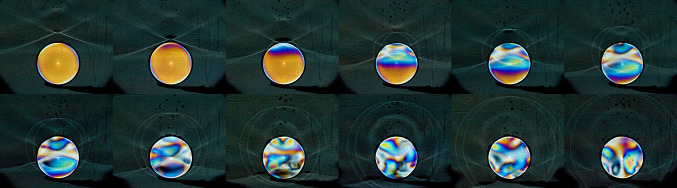
A shock wave approaches from above. It is partly reflected and transmitted through the dummy stone. Simultaneous photo-elastic imaging displays coloured lines of constant stress differences (qualitatively) within the sample (creation and copyright by the authors, previously published in Loske [7])

Shock wave impact on solid structures may be visualized using light transparent dummy stones. We used an acrylic cylinder of 2 cm length and a diameter of 15 mm positioned at right angle to the direction of shock wave propagation in the focal zone (Fig. 12).

The propagation velocity within the acrylic sample is faster than in the surrounding water. After internal reflection inside the sample a complex strain patterns all over the sample is visible. We could not recognize a circumferential squeezing effect as mandatory for quasi-static or dynamic squeezing. This might be due to the relatively small focal size of 3 mm, although Fig. 12 displays various shock wave fronts passing the sample laterally.

### (D) Cavitation

Cavitation bubbles have a significant impact on shock wave transmission if present in the propagation medium. Cavitation and gas bubbles absorb and scatter shock waves vigorously. They may diminish fragmentation efficiency when present in front of the shock wave. Usually, they vanish in less than a millisecond after shock wave passage. Within this time span, cavitation bubbles may be trapped at the stone surface and develop tiny and powerful micro-jets when collapsing asymmetrically. These micro-jets hit the stone surface with velocities of several hundred metres per second [13] and may erode the surface. This mechanism of cavitation is considered responsible for further reduction of fragments size as the second part of stone comminution after spallation and other effects (Zhong [2]).

**Fig. 13:**
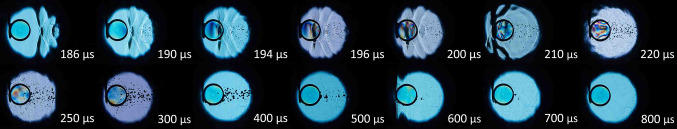
A series of shock waves approaching, entering and passing an acrylic cylinder, shock waves from right

Figure 13 shows a series of Schlieren and photo-elastic images of a acrylic cylinder at different points in time. Around 190 µs after generation the shock waves reach the cylinder and start to penetrate the surface (see e.g. Fig. 13/190 µs). Part of the wave front is reflected and runs in the reverse direction. The transmitted part exerts pressure and strain within the cylinder, shown by the colour change. Approximately 4 µs later (during the negative pressure phase) small cavitation bubbles start to grow. Some bubbles collapse after some microseconds whereas others keep growing some hundred microseconds to a maximum size of 2–2.5 mm (see e.g. Fig. 13/400 µs) after shock wave generation. Some of the bubbles stick at the cylinder surface until they collapse, presumably by generating a micro-jet towards the surface.

The cavitation bubble cloud around the sample (Ø 15 mm) extends to 15–20 mm and can not be made responsible for the pin-point-like erosion as displayed in Fig. 9.

We did not observe pitting like colour changes at the surface of the shocked acrylic cylinder induced by the impinging micro-jets. We expected to see pin-point-like pressure impacts generated by collapsing cavitation bubbles sticking to the surface. Although the pressure field of the focused shock is clearly visible within the sample, none of the images displayed small pitting like structures that might originate from impinging jets. In addition, fragmentation with model stones did not show the expected typical erosion pattern known from collapsing cavitation bubbles around the focus. A closer look showing more details may be necessary. After 800 µs of shock wave generation and approximately 600 µs after bubble generation all bubbles vanished.

## Discussion

The measurements described above elucidate the basic mechanism of interaction of a shock wave with an artificial stone without describing the fragmentation process in all details. Nevertheless, the principle mechanism bases on stringent application of Newton’s laws of inertia in an exemplary manner and is not limited to the specific conditions of our experiments. We may suppose the validity of Newton’s laws and the according mechanism for coarse as well as for fine fragments.

### Axial push and pull, a joggle mechanism and Hopkinson effect

Momentum transfer takes place within the positive part of the shock wave profile, which usually is in the range of 0.5–1 µs. During reflection, the positive amplitude of the incoming wave and the positive amplitude of the reflected wave superimpose to added pressure values higher than single pressure values. Decompression immediately after impact and reflection of the positive pressure curve together with the impinging negative tail of the shock wave generate tractive forces. Solid stone material, as solid bodies in general, resist much higher compressive forces than tensile forces [12]. Obviously, these forces are strong enough to extract small particles and dust from the surface, and catapult them against the direction of the impinging shock wave. We consider this mechanism responsible for erosion patterns at the front surface.

Push–pull, the antagonistic joggle action, provides the required mechanism for efficient fragmentation, both for coarse as well fine disintegration. According to the pressure profile of the impinging shock wave (see Fig. 1) accelerating and retracting forces successively act some microseconds (approximately 1–5 µs) one after the other but do not compensate completely. As shown above a resulting momentum pushes the target stone into the direction of the impinging shock wave. It moves the stone a little out of its rest position. This shift may be utilized to move fragments out of an unfavourable stone location such as lower kidney calices. After lithotripsy, fragments often remain in lower calices and resist stone removal procedures. They may be pushed by according well-directed shock waves in a controlled manner into a more favourable position such as the renal pelvis e.g. [14, 15].

Simultaneously, the Hopkinson effect creates flat spallation over the whole rear surface by splitting complete layers of stone material (Fig. 9c).

Joggle and Hopkinson effect are not restricted to specific focal dimensions, although a small focus provides a peaked load pattern with more inhomogeneous strain than a smooth pattern of a wide focus, probably provoking more internal cracks. The Hopkinson effect creates fragments (in our measurements approximately 3 mm, see Fig. 9b, c) limited by the pulse-length of the shock wave. The mechanism of momentum transfer has no lower limit size since inertia is a feature of small and large masses. However, for a given acceleration *a*, the inertia force (*F* = *ma*) is proportional to the accelerated mass and diminishes with smaller masses of fragments. Accordingly, there might be a limit due to material specific internal strength of the fragments resisting even exceptional acceleration.

Our experiments suggest a minor relevance of cavitation for stone disintegration. Zhong [2] differentiates between coarse and fine fragmentation requiring different mechanisms of fragmentation. The inertia model covers both, coarse and fine fragmentation based on the same mechanism of momentum transfer.

The inertia model depends on transmitted and reflected part of the momentum and accordingly varies with acoustic properties and shapes of the target stones, but does not require smooth surfaces or configurations to develop specific surface waves to generate crushing forces.

### Small or wide focus?

All experiments of this paper utilize a − 6dB-focus of 3 mm, which is considered a small focus compared to wide focal zones of e.g. the Dornier lithotripter HM3 with 12 mm [21] or the Xixin-lithotripter (XiXin Medical Instruments Co. Ltd, Suhou, China) with 18 mm lateral focal width [16].

There are numerous possibilities to improve shock wave lithotripsy. Tailoring the shock wave field to optimize fragmentation and minimize side effects is one of the preferred targets. The focal size in particular is one of the main topics [21].

The question of the optimal focal size seems to be answered in favour of a large focus, Eisenmenger et al. [16, 17], Rassweiler et al. [3], Chieh and Zhou [18]. Before discussing the issue we need to remind the definition of the − 6dB-focus. It confines the spatial area with pressure values higher than half the peak pressure, independent of the absolute value of the peak pressure [19]. If we consider a certain power level mandatory for effective shock wave treatment (treatment area for fragmentation or e.g. tissue stimulation), the spatial extension of this area may be concentric but is not determined by the − 6dB-focal extension. Depending on the selected power level, it may be smaller or larger [20]. Nevertheless, the terms “wide focus” and “small focus” are frequently used to characterize shock wave generators.

The paper of Eisenmenger et al. [16] published in 2002 aroused vigorous interest in wide-focus (18 mm) and low-pressure (< 30 MPa) lithotripsy. The idea was supported by the theory of shock wave squeezing as mechanism for stone fragmentation (Eisenmenger) [17].

The theory of quasi-static or dynamic squeezing requires an extended (wide) shock wave focus that passes the target stones laterally. High expectations in this technical idea motivated researchers to develop shock wave generators with extended focal zones (Chieh and Zhou [18]).

They made e.g. technical modifications on an electro-magnetic shock wave generator with an original smaller focus to increase focal size. According to the squeezing theory of Eisenmenger, the modification should have proven superior to the original small focus version. However, in order to reach equal or better results, a significant increase of the applied energy level was required. Escalation of focal energy usually increases fragmentation efficiency and is advantageous as long as side effects can be kept low and stones are not pushed out of their target position.

The original HM3 is still considered the Gold standard due to its wide focal zone of 12 mm (Evan et al. [21]). However, clinical results of various HM3-lithotripter studies were compiled and compared by Ogan and Pearl [22] showing similar results for several small focus devices with the exemption of the superb results of the original paper of Chaussy. Various newer publications report comparable results with smaller focus devices [23, 24].

Fifteen years after publication of Eisenmenger’s paper the idea of improving lithotripsy by wide-focus and low-pressure generators is still attractive [25] but up to our knowledge, further sound papers reporting superior clinical results of this technique are still missing.

We could show that a small focus of 3 mm provides the capacity to break artificial stones effectively and presented a novel view of a mechanism of fragmentation by inertia and momentum transfer. These findings question the idea of a wide focus as a mandatory requirement for superior fragmentation.

A benefit of the wide-focus device might reduce the targeting accuracy in case of dispersed fragments whereas a small focus may provide precisely focused stronger impacts on hard stones.

Alternative measures in particular how to handle a lithotripter seems to be of major importance [26, 27]. Coupling, targeting, treatment regime and others may be more important than focal size.

## Conclusion

The mechanism of shock wave fragmentation of brittle material as used in SWL is investigated based on momentum transfer and inertia phenomena. The enormous impulse on the target stone and the resulting acceleration in the direction of the impacting shock wave is strong enough to generate tensile forces to break stones. A locally confined impulse of the shock wave accelerates the stone and creates resisting inertia forces within the stone. Internal strain causes fracture lines and repeated impacts detach fragments from the weaker part of the stone, which are usually corners.

In case of the regular shaped plaster cube a specific fragmentation pattern occurs if the focal peak of the shock waves is centred at the middle of the front surface of the stone. The weakest areas, the front corners, fall off the stone without significant kinetic energy (Fig. 6). That means they stay almost stationary while the impulse of the shock wave pushes main part of the stone ahead.

Shock waves impact stones variably, depending on acoustic impedances, total mass of the stone material and shapes. The stones receive part of the acoustic energy provided by the shock wave, part of it is reflected and another part passes the stone. Momentum transfer and the according impulse (*F*Δ*t*) are strongest if the affected stone is massive and the mismatch of the acoustic impedance is large. This feature may explain why shock waves pass homogeneous tissue without significant lesion whereas they exert strong forces on stone material with different acoustic impedances.

We presented a novel mechanism of stone fragmentation based on Newton’s laws of momentum transfer and inertia, which does not require wide focal zones as in alternative mechanisms based on circumferential squeezing.

We are aware of the discrepancy between our findings and referred papers supporting a wide focus and would appreciate to start a lively debate on fragmentation mechanism.
